# Genetically predicted ankylosing spondylitis is causally associated with psoriasis

**DOI:** 10.3389/fimmu.2023.1149206

**Published:** 2023-07-06

**Authors:** Di Tian, Yuan Zhou, Yuting Chen, Ye Wu, Heng Wang, Chunchun Jie, Yan Yang, Yaoyao Liu, Haoyu Wang, Dian Zhou

**Affiliations:** ^1^ Department of Medical Service, The First Affiliated Hospital of Anhui Medical University, Hefei, China; ^2^ Department of Medical Service, The Second Affiliated Hospital of Anhui Medical University, Hefei, China; ^3^ Department of Epidemiology and Biostatistics, School of Public Health, Anhui Medical University, Hefei, Anhui, China; ^4^ Department of Human Resource, The First Affiliated Hospital of Anhui Medical University, Hefei, China; ^5^ School of Health Services Management, Anhui Medical University, Hefei, Anhui, China; ^6^ Party Committee Office, The Second Affiliated Hospital of Anhui Medical University, Hefei, China

**Keywords:** ankylosing spondylitis, psoriasis, Mendelian randomization, genome-wide association studies, single-nucleotide polymorphisms

## Abstract

**Background:**

Previous observational studies have reported the striking association between ankylosing spondylitis (AS) and psoriasis, but the causal relationship between the two diseases remains unclear.

**Methods:**

Two-sample Mendelian randomization (MR) analysis with methods of inverse-variance weighted, MR-Egger regression, weighted median, and weighted mode was conducted to evaluate the bidirectional causal associations between AS and psoriasis. Effective single-nucleotide polymorphisms (SNPs) from genome-wide association studies (GWAS) were selected as instrumental variables (IVs). Sensitivity analyses were also applied to verify whether heterogeneity and pleiotropy can bias the results.

**Result:**

We found positive causal effects of genetically increased AS risk on psoriasis (IVW: OR = 1.009, 95% CI = 1.005–1.012, *p* = 8.07E-07). Comparable outcomes were acquired by MR-Egger regression, weighted median, and weighted mode approaches. Nevertheless, we did not find significant causal effects of psoriasis on AS (IVW: OR = 1.183, 95% CI = 0.137–10.199, *p* = 0.879). The sensitivity analyses showed that the horizontal pleiotropy was unlikely to skew the causality. The leave-one-out analysis demonstrated that no single SNP can drive the MR estimates. No evidence of heterogeneity was found between the selected IVs.

**Conclusion:**

Our findings provide evidence that AS has positive causal effects on the risk of psoriasis in the European population.

## Introduction

Ankylosing spondylitis (AS) is an immune-related chronic inflammatory disorder, which mainly involves the spine and the sacroiliac joints and arouses pain and stiffness of these joints (1). AS affects approximately 0.1%–0.5% of the general population worldwide (2). Although the nosogenesis of AS remains largely unknown, numerous evidence has revealed that the interactions of genetic inheritance, environmental exposure, chronic infections, and abnormal endocrine contribute to the occurrence and development of the disease (3, 4).

Psoriasis is a common inflammatory papulosquamous skin disorder, which is caused by the interplay of genetic, immunological, and environmental factors with a wide range of clinical manifestations (5, 6). Psoriasis can occur at any age and is associated with several comorbidities, such as lymphoma and depression (7). WTO recognized psoriasis as a “chronic, non-communicable, painful, disfiguring, and disabling disorder for which there is no cure” (8). It has been estimated that over 60 million adults and children around the world are affected by psoriasis, imposing a huge burden on individuals and society (8).

The parallels between AS and psoriasis has long been recognized. For both diseases, heritability is the main risk determinant, and inflammatory cytokines, such as interleukin (IL)-17, IL-23, and tumor necrosis factor (TNF)-α are key drivers, indicating that they might share similar pathogenesis (9, 10). There is a striking association between AS and psoriasis. A variety of cross-sectional and cohort studies reported that AS patients were more likely to develop psoriasis than heathy controls (11, 12). The prevalence of psoriasis in AS was estimated at 9.3% according to a previous meta-analysis (13). Moreover, it has been suggested that psoriasis may predate the onset or diagnosis of AS (14). Studies have reported that patients with AS combined with psoriasis have higher disease activity scores (15). Notably, the hallmarks of psoriasis are the inflammation of skin and joints (16). Nevertheless, the causal relationship between the two conditions is still ambiguous, which warrants further research to increase the current understanding of the pathogeneses of these two diseases.

Mendelian randomization (MR) is a developing popular approach, which usually serves genetic variants as instrumental variables (IVs) to infer whether there is causal relationship between the exposure and the outcome (17). Since the genetic variations are stochastically arranged in meiosis and fixed after fertilization, this technology enables to conquer several shortcomings of traditional observational studies, including the interference of confounding factors, reverse causation, and selection biases.

In this current study, a bidirectional two-sample MR analysis was conducted, in order to determine whether there is a significant causal relationship between AS and psoriasis risk.

## Materials and methods

### Data sources

Single-nucleotide polymorphisms (SNPs), which were selected from the publicly available genome-wide association studies (GWAS) database, were severed as IVs for exposure (AS) and outcome (psoriasis). No additional ethical statement or consent was therefore required. SNPs that associated with AS were extracted from a large AS GWAS conducted by the International Genetics of Ankylosing Spondylitis Consortium (IGAS), including 9,069 cases and 13,578 controls of European ancestry (https://gwas.mrcieu.ac.uk/datasets/ebi-a-GCST005529) (18). The diagnosis of AS was based on the revised New York criteria of 1984 (19). The summary-level data on the effect of the AS-associated SNPs on psoriasis were derived from another independent GWAS by Neale laboratory, which involved 3,871 cases and 333,288 controls of European ancestry (https://gwas.mrcieu.ac.uk/datasets/ukb-a-100). Psoriasis was diagnosed by professional physicians through pattern recognition that was careful morphologic assessment of the skin lesions (20).

### SNP selection

To guarantee the correctness and truth of the conclusions on the causality between AS and psoriasis risk, a series of quality control steps were carried out to select effective IVs. We selected the SNPs with a genome-wide significant association (*p* < 5E-08), and without linkage disequilibrium (LD) (*r^2^* < 0.001, clumping distance = 10,000 kb) in summary statistics. When the exposure-related SNPs were not present in the outcome dataset, the proxy SNPs significantly correlated with the SNPs of interest were used (*r^2^* > 0.8). To make sure that the impact of SNPs on exposure corresponds to the same allele as the impact on the outcome, palindromic SNPs with intermediate allele frequencies were not included in IVs. SNPs with the minor allele frequency (MAF) less than 0.01 were also excluded. Finally, eligible SNPs were selected as valid IVs for the final model.

### MR assumptions

**Figure 1:**
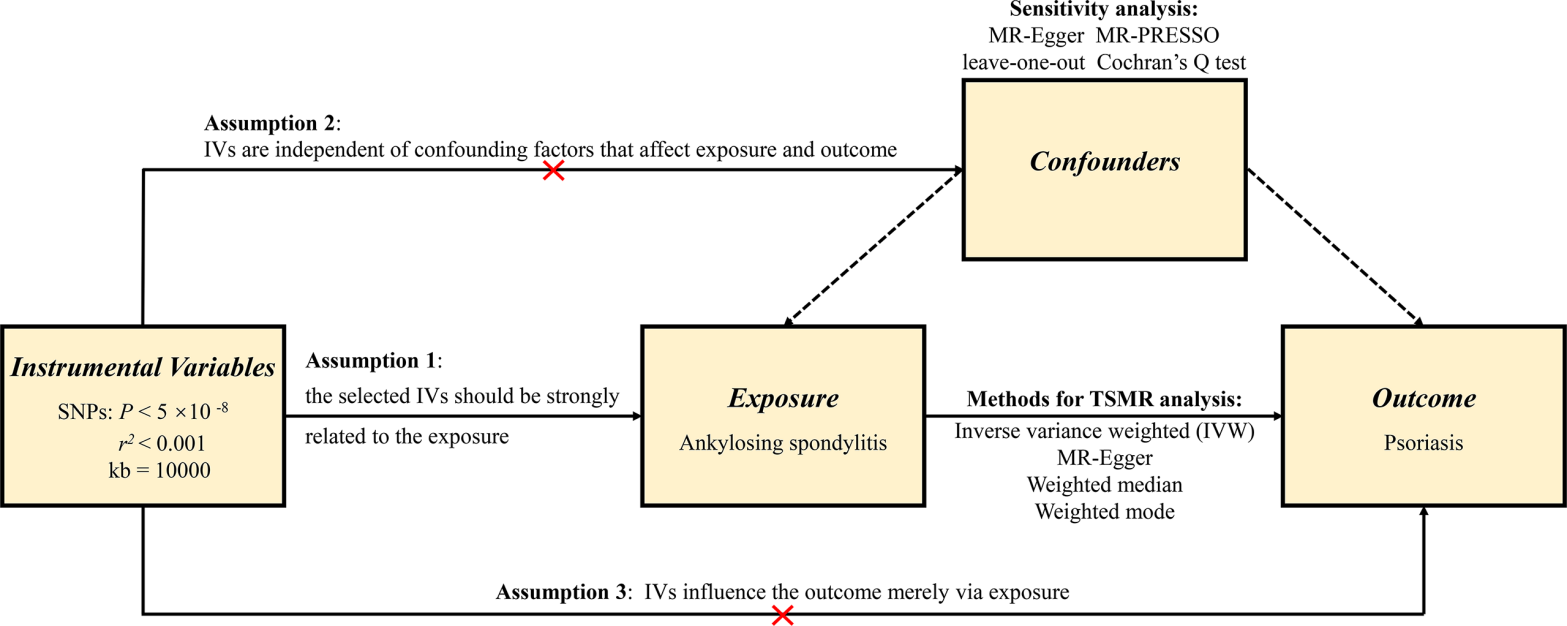
An overview of this Mendelian randomization (MR) study design.

In order to minimize the bias on the outcomes, the MR analysis should comply with three pivotal assumptions (21). First, the IVs that were selected must be robustly related to the exposure. In the current study, the strength of the association between the IVs and the exposure was assessed by *F* value, which is expressed as *R* ^2^ (*n−k−*1)*/k* (1*−R^2^*) (*R^2^*: the cumulative interpreted variance of chosen SNPs on the exposure, *k*: the number of chosen SNPs, *n*: the sample size). If *F* is greater than 10, the correlation is regarded as strong enough and enable to avert bias brought by weak IVs. Second, IVs need to be independent of confounding factors that can affect both exposure and outcome. Third, IVs should affect the outcome merely by exposure, which requires that there is no horizontal pleiotropy effect between the IVs and outcome. **Figure 1** shows an overview of the study design.

### Effect size estimate

We performed two-sample MR analysis to evaluate the causal relationship between exposure (AS) and outcome (psoriasis). Multiple complementary methods were applied, including the inverse variance weighted (IVW), the MR-Egger regression, the weighted median, and the weighted mode approaches. IVW is constitutionally a meta-analysis method, which requires all IVs to be valid and combines the Wald ratios of the causal effects of each SNP. In the absence of horizontal pleiotropy, IVW is perceived as the primary method because it provides the most accurate estimates (22). MR-Egger regression conducts a weighted linear regression and produces consistent causal estimates, even if the selected IVs are all invalid. Nevertheless, its accuracy is relatively low (23). The weighted median supplies reliable effect estimates of causal effects when less than 50% of the weight in the analysis comes from invalid IVs (24). The weighted median method possesses several important advantages over the MR-Egger regression, since it has lower type I error and higher causal estimate power. Additionally, the weighted mode approach estimates the causal effects of the subset with the maximum number of SNPs by clustering SNPs into subsets with similar causal effects (25).

### Sensitivity analysis

To rule out possible breaches of the MR assumptions, multiple sensitivity analyses were conducted to verify whether pleiotropy and heterogeneity in the genetic instruments tested could bias the MR outcomes. Pleiotropy is the phenomenon that a single locus influences plural phenotypes. Horizontal pleiotropy occurs when a genetic variable is correlated with more than one phenotype by separatory pathways, which can distort the outcomes of MR analysis. Herein, we performed the MR-Egger regression and evaluated the intercept, in order to detect as well as adjust for the potential horizontal pleiotropic effects within the selected IVs (assumption 3) (26). The MR pleiotropy residual sum and outlier (MR-PRESSO) test was also performed to detect pleiotropy, exclude outliers, and reappraise the effect estimates (assumption 3) (27). Cochran’s *Q* statistic was used in order to quantify the heterogeneity within the selected IVs (assumption 2) (28). To make sure that the MR conclusions are not affected by certain IVs, the leave-one-out sensitivity analysis was performed by removing each SNP in turn when conducting the MR analysis (assumption 2).

A two-sided *p*-value lower than 0.05 was considered as statistically significant. All data analyses were completed by the “TwoSampleMR” and “MRPRESSO” packages of the R 4.2.2 software (www.r-project.org).

## Results

### Genetic instruments selection

Initially, a total of 1,036 genome-wide significant SNPs were identified (*p* < 5E-08) from the GWAS study conducted by Cortes et al. (18). After removing SNPs that had LD effects or were not available in the summary statistic of outcome, 16 SNPs explaining 1.85% of the variance for AS were selected as IVs for the further two-sample MR analysis. The detailed information of these SNPs including effect allele, other allele, beta, SE, and *p*-value are summarized in **Supplementary Table S1**. There was no LD (*r* ^2^ < 0.001) between these SNPs, and the *F* statistics were all greater than 10 with the average *F* value of 134.872 for AS, indicating that the selected IVs conformed to the intense correlation assumption of MR, and the instrument bias was weak, which cannot significantly affect the estimation of MR.

### Two-sample MR analysis

**Figure 2:**
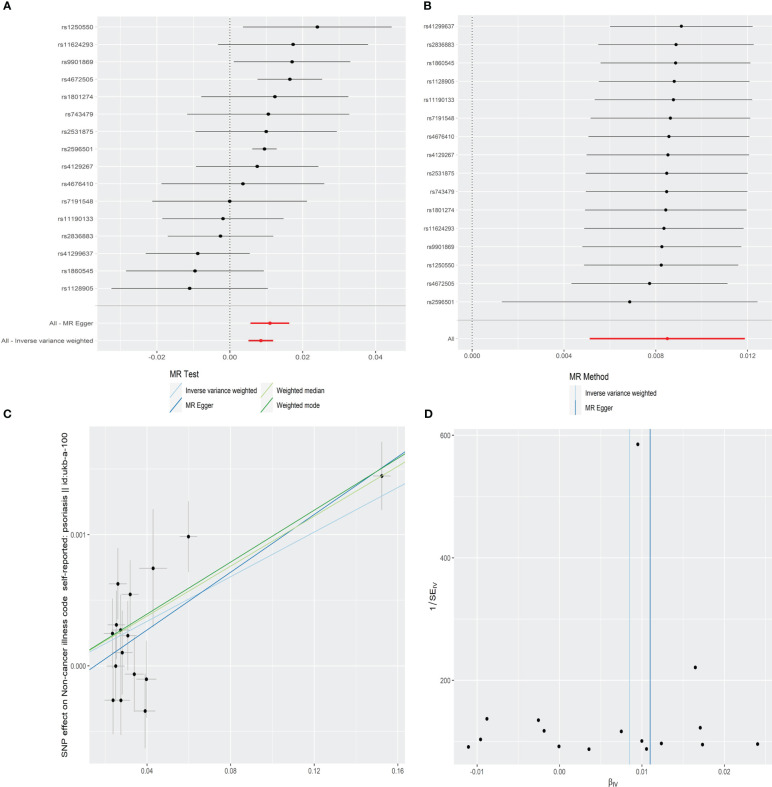
Forest plot **(A)**, sensitivity analysis **(B)**, scatter plot **(C)**, and funnel plot **(D)** of the effect of AS on psoriasis.

The IVW result demonstrated that the per-unit increase in the log-odds of developing AS was significantly associated with an increased risk of having psoriasis at *p* less than 0.05 (OR = 1.009, 95% CI = 1.005–1.012, *p* = 8.07E-07). In addition, MR-Egger (OR = 1.011, 95% CI = 1.006–1.016, *p* = 1.19E-03), weighted median (OR = 1.010, 95% CI = 1.006–1.013, *p* = 2.14E-09), and weighted mode (OR = 1.010, 95% CI = 1.007–1.013, *p* = 2.90E-05) also provided consistent results that AS had a positive causal effect on psoriasis risk (**Table 1** and **Figure 2A**).

**Table 1:** MR estimates from each method of assessing the causal effects of AS on psoriasis risk.

Exposure/outcome	Number of SNPs	Methods	*β*	SE	OR (95% CI)	*p*-value	Horizontal pleiotropy			Heterogeneity	
							Egger intercept	SE	*p*-value	Cochran’s *Q*	*p*-value
AS/ Psoriasis	16	MR-Egger	0.011	0.003	1.011 (1.006–1.016)	1.19E−03	−0.0002	0.0001	0.257	22.350	0.072
		Inverse-variance weighted	0.008	0.002	1.009 (1.005–1.012)	8.07E−07					
		Weighted median	0.010	0.002	1.010 (1.006–1.013)	2.14E−09					
		Weighted mode	0.010	0.002	1.010 (1.007–1.013)	2.90E−05					

AS, ankylosing spondylitis; CI, confidence interval; MR, Mendelian Randomization; OR, odds ratio; SNP, single-nucleotide polymorphism; SE, standard error.

p < 0.05 was considered statistically significant.

The leave-one-out sensitivity analysis showed that there was no single SNP driving the MR estimates (**Figure 2B**). Furthermore, the horizontal pleiotropy between IVs and outcome was evaluated by MR-Egger regression, and the result suggested that the horizontal pleiotropy was unlikely to skew the causal relationship between AS and psoriasis (intercept = −0.0002, SE = 0.0001, *p* = 0.257) (**Table 1** and **Figure 2C**). The MR-PRESSO global test also found no outlier SNPs or a horizontal pleiotropic effect of AS on the risk of psoriasis (*p* = 0.115). Cochran’s *Q* test observed no significant heterogeneity among the selected IVs (*Q* = 22.350, *p* = 0.072) (**Table 1** and **Figure 2D**).

### Further analyses

**Figure 3:**
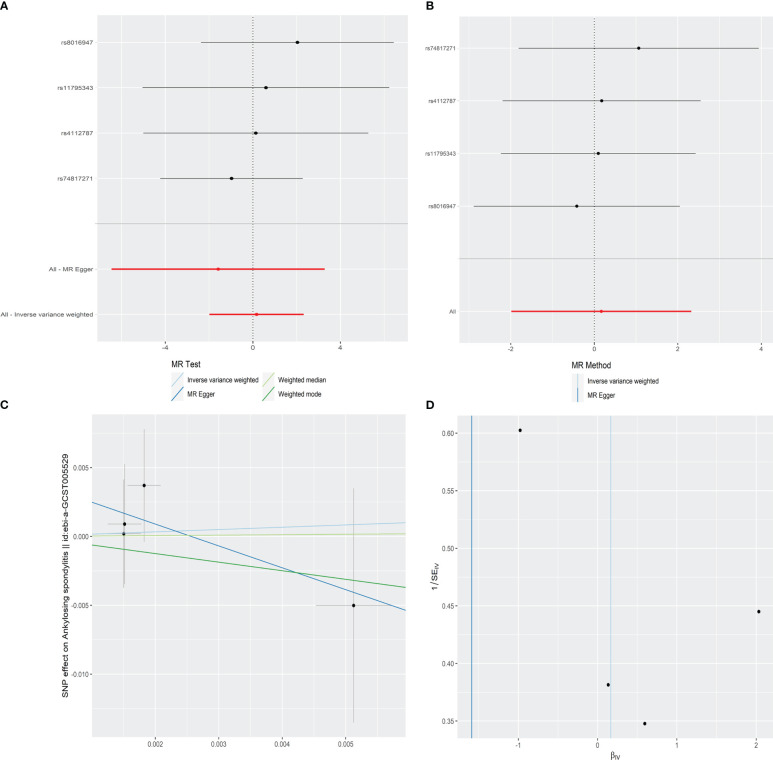
Forest plot **(A)**, sensitivity analysis **(B)**, scatter plot **(C)**, and funnel plot **(D)** of the effect of psoriasis on AS.

In order to explore the causal effect of psoriasis on AS risk, we further carried out the two-sample MR analysis by using psoriasis as exposure and AS as outcome. There were 21 significant and independent SNPs (*p* < 5E-08, *r* ^2^ < 0.001) correlated with the risk of psoriasis, which were derived from the large-scale psoriasis GWAS by the Neale laboratory. However, 17 SNPs were excluded because of no corresponding outcomes in AS. Thus, four SNPs were finally selected as the IVs for the MR analysis (**Supplementary Table S2**). The *F* statistics of these SNPs were all larger than 10 with the average *F* value of 46.406 for psoriasis, indicating the absence of weak IV bias. According to IVW, MR-Egger, weighted median, and weighted mode approaches, there was no evidence showing causal association of an increased risk of psoriasis with the change in the risk of AS (**Table 2** and **Figure 3A**). Sensitivity analysis using the leave-one-out method also proved no significant causal relationship of the psoriasis and AS risk (**Figure 3B**). No significantly horizontal pleiotropy was detected by MR-Egger regression (intercept = 0.0041, SE = 0.0052, *p* = 0.513) (**Table 2** and **Figure 3C**). The MR-PRESSO global test also observed no outlier SNPs or a horizontal pleiotropic effect of psoriasis on the risk of AS (*p* = 0.718). Additionally, Cochran’s *Q* test indicated that there was no significant heterogeneity across estimates of included SNPs (MR-Egger: *Q* = 0.567, *p* = 0.753) (**Table 2** and **Figure 3D**).

**Table 2:** MR estimates from each method of assessing the causal effects of psoriasis on AS risk.

Exposure/ outcome	Number of SNPs	Methods	*β*	SE	OR (95% CI)	*p*-value	Horizontal pleiotropy			Heterogeneity	
							Egger intercept	SE	*p*-value	Cochran’s *Q*	*p*-value
Psoriasis /AS	4	MR-Egger	−1.588	2.485	0.204 (0.002–26.653)	0.588	0.0041	0.0052	0.513	0.567	0.753
		Inverse-variance weighted	0.168	1.099	1.183 (0.137–10.199)	0.879					
		Weighted median	0.033	1.266	1.033 (0.086−12.352)	0.979					
		Weighted mode	−0.622	1.608	0.537 (0.023−12.553)	0.725					

AS, ankylosing spondylitis; CI, confidence interval; MR, Mendelian Randomization; OR, odds ratio; SNP, single-nucleotide polymorphism; SE, standard error.

p < 0.05 was considered statistically significant.

## Discussion

This is the first study using MR method and integrating large-scale GWAS data to investigate the bi-directional causal relationship between AS and psoriasis. Our findings implied that AS had positive causal effects on the risk of psoriasis, but did not show that psoriasis had positive causal effects on the risk of AS in individuals from European descent. Suffering from AS would be the causal element of the increased risk of psoriasis, indicating that the two diseases might share similar pathogenesis.

AS and psoriasis are featured by disturbances in systemic or organ-specific immune system regulatory pathways that lead to uncontrolled inflammation. These “seronegative” disorders are clinically closely related and often co-occur in both patients and families. Despite the fact that the exact mechanisms linking AS and psoriasis are not fully explained, the hyperactivity of the IL-17/IL-23 cytokine axis was proposed to account for the pathogenic association (29, 30). Numerous studies have demonstrated increased levels of IL-17 and IL-23 in the serum and synovium of AS patients, as well as the skin of psoriasis patients (31, 32). Inhibitors targeting IL-17 have shown positive effects in the treatment of AS and psoriasis (33, 34). Furthermore, the overlap between the two diseases in genetically identified pathogenic pathways has also been verified, such as the polymorphisms of ERAP1 and HLA-class I genes (35, 36).

The high risk of psoriasis in AS patients has been observed in previous epidemiological studies. In a large retrospective cohort study conducted using data from the Clinical Practice Research Datalink (CPRD) during 1987–2012, Stolwijk et al. (11) demonstrated that 4.4% of patients had psoriasis at the time of diagnosis of AS, and the incidence of psoriasis was 3.4 per 1,000 person-years. Furthermore, with the 12-year follow-up data from the Outcome in Ankylosing Spondylitis International Study (OASIS), Essers et al. (37) found that psoriasis was present in 4.1% of the 216 AS patients at baseline and psoriasis was not associated with longer disease duration. Hence, they speculated that psoriasis may already be present before the onset or diagnosis of AS (37). Given the presence of confounders in observational studies, it is unable to determine which comes first, the psoriasis or AS, and whether there is a causal relationship between the two diseases. The findings of our study provided new information on it. Briefly, AS had a causal effect on psoriasis, but psoriasis was not the cause of AS, which provided a theoretical basis for paying attention to skin involvement in AS patients.

The causal effect of AS on psoriasis is of great significance for the diagnosis, treatment and prognosis of AS. AS has the longest delay in diagnosis among rheumatic diseases, ranging from 5 to 10 years, because the diagnostic criteria of AS depend on radiographic sacroiliitis, which usually appears in the late stage of the disease (38). The presence of psoriasis increases the probability of AS in patients with chronic back pain and may contribute to the diagnosis of the disease. Furthermore, there are conflicting results regarding the impact of psoriasis on clinical manifestations and disease severity in AS patients. Several studies showed that AS patients with concomitant psoriasis had more severe disease course than patients with primary AS (15, 39). However, another research reported that there was no difference in disease phenotype between AS patients with or without psoriasis (40). Despite no consensus has been reached, there is no doubt that AS patients with concomitant psoriasis adds to the complexity of care, influences treatment decisions and patients’ quality of life, and requires cooperation with dermatologists. The results of our study also underlined the significance of preventing and managing psoriasis in patients with AS.

Several disadvantages should be noticed in the present MR study. Firstly, the data we used belonged to two large-scale GWAS analyses, so that detailed demographic information was lacking, and we cannot conduct any stratification analyses to adjusted for other covariables. Secondly, the study focused on European ancestry, so it is limited to extrapolate the results to other ethnics. Thirdly, the degree of sample overlap between the exposure and outcome is difficult to estimate. Finally, there is a lack of non-radiographic axial population, so it is unable to provide new information about whether AS and non-radiographic axial spondyloarthritis are genetically different.

Despite these disadvantages, our study also has several strengths. Firstly, this is the first MR research to investigate the causal relationship between AS and psoriasis with a large sample size. Secondly, the interference of confounding factors on results and reverse causality has been overcome by using the design of MR approach.

As a whole, this MR study suggests that AS has positive causal effects on the risk of psoriasis, but psoriasis does not have positive causal effects on the risk of AS. Further studies with updated data from large genetic research are required to confirm the findings of this study.

## Data availability statement

The original contributions presented in the study are included in the article/**Supplementary Material**. Further inquiries can be directed to the corresponding author.

## Author contributions

DT: Conceptualization, Methodology, Software, Writing-Original draft preparation; YZ: Data curation, Visualization, Investigation; YC, YW, HW, and CJ: Supervision, Software; YY, YL, and HYW: Validation; DZ: Writing- Reviewing and Editing. All authors contributed to the article and approved the submitted version.
