# Supplementary material for: Genetically predicted ankylosing spondylitis is causally associated with psoriasis
Source: Front Immunol. 2023 Jul 6;14:1149206. doi: 10.3389/fimmu.2023.1149206 (PMC10357290; doi:10.3389/fimmu.2023.1149206)
Supplement: Supplementary file 1 [file DataSheet_1.docx]

Supplementary Materials

**Table S1.** Detailed information of instrumental variables utilized in the MR analysis of the causal effects of AS on psoriasis risk.

| **SNP** | **Chr** | **Pos** | **Effect allele** | **Other allele** | **AS** | | | |  | **Psoriasis** | | |
| --- | --- | --- | --- | --- | --- | --- | --- | --- | --- | --- | --- | --- |
|  |  |  |  |  | ***β*** | **SE** | ***P* value** | ***F* statistic** |  | ***β*** | **SE** | ***P* value** |
| Instrumental variables of AS | | |  |  |  |  |  |  |  |  |  |  |
| rs11190133 | 10 | 101278725 | T | C | -0.034 | 0.004 | 4.84E-14 | 56.792 |  | 0.000 | 0.000 | 0.826 |
| rs1128905 | 9 | 139253839 | C | T | -0.024 | 0.004 | 6.95E-09 | 33.549 |  | 0.000 | 0.000 | 0.313 |
| rs11624293 | 14 | 88488821 | C | T | 0.043 | 0.007 | 1.49E-10 | 41.040 |  | 0.001 | 0.000 | 0.099 |
| rs1250550 | 10 | 81060317 | A | C | -0.026 | 0.004 | 1.46E-09 | 36.582 |  | -0.001 | 0.000 | 0.021 |
| rs1801274 | 1 | 161479745 | G | A | 0.025 | 0.004 | 1.35E-09 | 36.741 |  | 0.000 | 0.000 | 0.231 |
| rs1860545 | 12 | 6446777 | A | G | -0.027 | 0.004 | 2.78E-10 | 39.821 |  | 0.000 | 0.000 | 0.321 |
| rs2531875 | 17 | 26148167 | T | G | -0.027 | 0.004 | 1.22E-10 | 41.437 |  | 0.000 | 0.000 | 0.313 |
| rs2596501 | 6 | 31321211 | T | C | -0.152 | 0.004 | 1.00E-200 | 1319.308 |  | -0.001 | 0.000 | 0.000 |
| rs2836883 | 21 | 40466744 | A | G | -0.040 | 0.005 | 6.46E-17 | 69.832 |  | 0.000 | 0.000 | 0.729 |
| rs4129267 | 1 | 154426264 | T | C | -0.031 | 0.004 | 3.32E-13 | 53.008 |  | 0.000 | 0.000 | 0.382 |
| rs41299637 | 1 | 200877850 | G | T | -0.039 | 0.005 | 1.81E-15 | 63.259 |  | 0.000 | 0.000 | 0.227 |
| rs4672505 | 2 | 62560332 | G | A | -0.060 | 0.004 | 5.14E-47 | 207.374 |  | -0.001 | 0.000 | 0.000 |
| rs4676410 | 2 | 241563739 | A | G | 0.028 | 0.005 | 9.90E-09 | 32.861 |  | 0.000 | 0.000 | 0.754 |
| rs7191548 | 16 | 28614734 | C | T | 0.025 | 0.004 | 5.51E-09 | 34.000 |  | 0.000 | 0.000 | 0.995 |
| rs743479 | 21 | 45611950 | T | C | -0.023 | 0.004 | 2.03E-08 | 31.465 |  | 0.000 | 0.000 | 0.353 |
| rs9901869 | 17 | 45575206 | A | G | 0.032 | 0.004 | 6.04E-15 | 60.890 |  | 0.001 | 0.000 | 0.036 |

AS, ankylosing spondylitis; Chr, chromosome; MR, Mendelian Randomization; Pos, position; SNP, single nucleotide polymorphism; SE, standard error.

**Table S2.** Detailed information of instrumental variables utilized in the MR analysis of the causal effects of psoriasis on AS risk.

| **SNP** | **Chr** | **Pos** | **Effect allele** | **Other allele** | **Psoriasis** | | | |  | **AS** | | |
| --- | --- | --- | --- | --- | --- | --- | --- | --- | --- | --- | --- | --- |
|  |  |  |  |  | ***β*** | **SE** | ***P* value** | ***F* statistic** |  | ***β*** | **SE** | ***P* value** |
| Instrumental variables of psoriasis | | |  |  |  |  |  |  |  |  |  |  |
| rs11795343 | 9 | 32523737 | C | T | -0.002 | 0.000 | 1.16E-08 | 32.553 |  | -0.001 | 0.004 | 0.836 |
| rs4112787 | 1 | 152551325 | T | C | 0.002 | 0.000 | 4.35E-08 | 29.987 |  | 0.000 | 0.004 | 0.959 |
| rs74817271 | 5 | 150469973 | A | G | 0.005 | 0.001 | 6.30E-18 | 74.432 |  | -0.005 | 0.009 | 0.556 |
| rs8016947 | 14 | 35832666 | G | T | 0.002 | 0.000 | 3.06E-12 | 48.651 |  | 0.004 | 0.004 | 0.366 |

AS, ankylosing spondylitis; Chr, chromosome; MR, Mendelian Randomization; Pos, position; SNP, single nucleotide polymorphism; SE, standard error.
